# The QUILT study: quilting sutures in patients undergoing breast cancer surgery: a stepped wedge cluster randomized trial study

**DOI:** 10.1186/s12885-023-11154-0

**Published:** 2023-07-17

**Authors:** L. J. van Zeelst, B. ten Wolde, J. D. J. Plate, J. H. Volders, R.R.J.P. van Eekeren, A. Doeksen, M. L. Hoven-Gondrie, A. F. T. Olieman, Y. E. A. van Riet, A. P. Schouten van der Velden, S. Vijfhuize, H. H. G. Witjes, J. H. W. de Wilt, L. J. A. Strobbe

**Affiliations:** 1 Department of Surgical Oncology, Canisius Wilhelmina Hospital, Weg Door Jonkerbos 100, 6532 SZ Nijmegen, The Netherlands; 2 Department of Surgical Oncology, Diakonessenhuis, Bosboomstraat 1, 3582 KE Utrecht, The Netherlands; 3 Department of Surgical Oncology, Rijnstate Hospital, Wagnerlaan 55, 6815 AD Arnhem, The Netherlands; 4 Department of Surgical Oncology, St. Antonius Hospital, Soestwetering 1, 3543 AZ Nieuwegein, Netherlands; 5 Deparment of Surgical Oncology, Hospital Gelderse Vallei, Willy Brandtlaan 10, 6716 RP Ede, Netherlands; 6 Department of Surgical Oncology, Martini Hospital, Van Swietenplein 1, 9728 NT Groningen, Netherlands; 7 Department of Surgical Oncology, Catharina Hospital, Michelangelolaan 2, 5623 EJ Eindhoven, Netherlands; 8 Department of Surgical Oncology, St. Jansdal Hospital, Wethouder Jansenlaan 90, 3844 DG Harderwijk, Netherlands; 9 Deparment of Surgical Oncology, Bravis Hospital, Boerhaavelaan 25, 4708 AE Roosendaal, Netherlands; 10 Department of Surgical Oncology, Onze Lieve Vrouwe Gasthuis, Jan Tooropstraat 164, 1061 AE Amsterdam, Netherlands; 11 Radboudumc Department of Surgical Oncology, Geert Grooteplein Zuid 10, 6525 GA Nijmegen, Netherlands

**Keywords:** Mastectomy, Breast cancer surgery, Quilting, Flap fixation, Seroma, Complications, Shoulder function, Pain, Cosmetic outcome, Health care consumption

## Abstract

**Background:**

Seroma is the most common complication following breast cancer surgery, with reported incidence up to 90%. Seroma causes patient discomfort, is associated with surgical site infections (SSI), often requires treatment and increases healthcare consumption. The quilting suture technique, in which the skin flaps are sutured to the pectoralis muscle, leads to a significant reduction of seroma with a decrease in the number of aspirations and surgical site infections. However, implementation is lagging due to unknown side effects, increase in operation time and cost effectiveness. Main objective of this study is to assess the impact of large scale implementation of the quilting suture technique in patients undergoing mastectomy and/or axillary lymph node dissection (ALND).

**Methods:**

The QUILT study is a stepped wedge design study performed among nine teaching hospitals in the Netherlands. The study consists of nine steps, with each step one hospital will implement the quilting suture technique. Allocation of the order of implementation will be randomization-based. Primary outcome is ‘textbook outcome’, i.e.no wound complications, no re-admission, re-operation or unscheduled visit to the outpatient clinic and no increased use of postoperative analgesics. A total of 113 patients is required based on a sample size calculation. Secondary outcomes are shoulder function, cosmetic outcome, satisfaction with thoracic wall and health care consumption. Follow-up lasts for 6 months.

**Discussion:**

This will be one of the first multicentre prospective studies in which quilting without postoperative wound drain is compared with conventional wound closure. We hypothesize that quilting is a simple technique to increase textbook outcome, enhance patient comfort and reduce health care consumption.

## Introduction

### Background and rationale {6a}

The most frequently reported complication after mastectomy and/or axillary lymph node dissection (ALND) is seroma formation. Seroma is a collection of any fluid underneath the skin flaps or in the axillary space after surgery. Seroma incidence following mastectomy is reported to be up to 90% [1–3]. Seroma causes patient discomfort (pain, impaired shoulder mobility, swelling, fear of recurrence), often urging for needle aspirations. This can result in a site of entry for bacteria causing surgical site infections, wound complications and eventually potential delay to adjuvant therapy. It also leads to an increased healthcare consumption due to prolonged hospital stay, more outpatient clinic visits and re-operations [1, 3–7].

Seroma is a much discussed topic in literature and preventive measures are extensively reported. Fibrin glue, bovine thrombin, external compression and shoulder immobilization all seem to be ineffective or at best moderately effective [8–11]. Closure of the dead space after surgery seems to be a key factor in reducing seroma formation [3]. The quilting suture technique, where the skin flaps are fixated to the pectoralis major muscle, decreases the incidence of seroma formation. Quilting is a technique regularly used following flap harvesting in autologous breast reconstruction [12, 13]. Quilting following primary breast cancer surgery is reported increasingly over the past years [6, 14–16]. In studies comparing quilting to the conventional closure, seroma incidence decreased from 81 to 23% [6] and 23 to 8% [17] due to quilting. Also the number of aspirations, volume of aspirations and incidence of surgical site infections, wound edge necrosis, hematoma and re-operations decreased [5–7, 18, 19].

In the vast majority of hospitals mastectomy incision is closed at subcutaneous and skin level over a vacuum drain. Recent evidence, however, suggests that a postoperative drain has no value in reducing seroma formation in combination with the quilting suture technique following mastectomy [7, 20–22].

In addition to diminishing patient discomfort, quilting could decrease healthcare consumption. The omission of drains and the limited complication risk should facilitate the evolution of breast cancer surgery to day care treatment. Seroma related visits to the outpatient clinic, re-admissions and complication related to surgery are likewise expected to decrease.

Despite the first report of success from quilting sutures in 1993, this technique is still not embedded in global daily practice [23]. Surgeons are reluctant to implement the quilting technique because of hypothetical disadvantages of quilting. In the absence of well-founded studies, critics are raised such as increased postoperative pain and decreased shoulder function caused by tight sutures, concerns exist about cosmetic outcome and possible skin dimpling and longer operating time could give raise to increased healthcare costs [16, 24].

To date, no multicenter prospective studies have been published in which quilting without postoperative wound drain is compared with conventional wound closure with postoperative wound drain, reporting on seroma and other potential (dis)advantages.

The objective of this study is therefore, to assess the impact of quilting in patients undergoing mastectomy and/or ALND in nine teaching hospitals. Postoperative complications, shoulder function, cosmetic outcome, satisfaction with thoracic wall, postoperative pain and healthcare consumption of patients treated with the quilting suture technique without drain will be compared with conventional wound closure. All these will be summarized in the primary outcome as textbook outcome. Textbook outcome is achieved if a patient suffers no wound complications, requires no re-admissions or re-operation in relation to primary surgery nor any unscheduled visit to the outpatient clinic and the postoperative use of analgesics after six months is not increased compared to pre-operative.

Textbook outcome is defined as the percentage of patients for whom all desired short-term outcomes of care are realized [25]. This outcome provides a meaningful summary measure of health care quality for patients, providers and insurance companies. It is a useful outcome to make a comparison between different hospitals. Usually significant variability between hospitals can be expected, all scoring well on different single factors, but poorly in other factors. Besides, a compound indicator reflecting the ideal surgical outcome is more meaningful than one single outcome item and it is a useful tool for managing patient expectations [26].

### Objectives {7}

The objective is to report whether implementation of quilting in breast cancer surgery improves textbook outcome. Data is collected on the incidence of seroma and other postoperative complications, postoperative pain, shoulder function and health care consumption.

We hypothesize that implementation of quilting in breast cancer patients undergoing mastectomy and/or an axillary lymph node dissection increases textbook outcome. We expect incidences of seroma and other postoperative wound complications to decrease, with the same cosmetic outcome and without an increase in postoperative pain or impairing shoulder function. Moreover we hypothesize health care costs to reduce as a result of quilting.

### Study design {8}

The QUILT study is a stepped wedge cluster randomized study.

## Methods: Participants, interventions and outcomes

### Study setting {9}

**Fig. 1:**
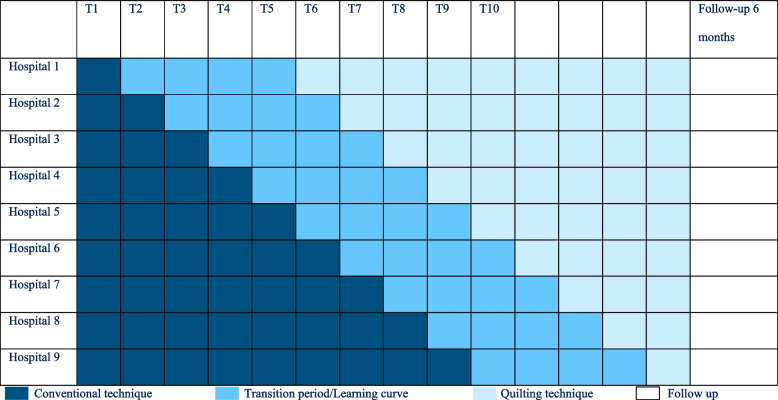
Stepped wedge schedule for the QUILT study

The organizing hospital, Canisius Wilhelmina Hospital, is excluded from participation because the quilting suture technique already is standard care for multiple years. The participating nine hospitals provide standard care by conventional wound closure with postoperative drainage. These nine hospitals will be randomized for implementation of the quilting suture technique as a cluster. With each step every two weeks, one cluster is randomised to implement the quilting suture technique, there will be nine steps. Transition period or learning curve is expected to be 8 weeks. Follow up continues for six months postoperatively (Fig. 1). At T1 all hospitals start including patients.

### Eligibility criteria {10}

#### Inclusion criteria

- all patients > 18 years of age undergoing mastectomy and/or axillary lymph node dissection
- be irrespective of the nature of the primary tumour: prophylactic, risk reducing, benign, in situ carcinoma and invasive primary or recurrent carcinoma will be eligible, irrespective of preoperative systemic therapy.

#### Exclusion criteria

- patients who objected to participation
- mentally incompetent patients or otherwise unable to complete a questionnaire
- immediate breast reconstruction
- pregnancy

### Who will take informed consent? {26a}

Quilting, or skin flap fixation, is an accepted suturing technique. Pre-operatively at the outpatient clinic, patients will be informed about the study by their treating surgeon or breast care nurse. In addition, an instruction is handed out on how to object to participation in the study if patients do not wish to receive questionnaires, object to have pictures taken or object to their medical data being processed for this study purpose.

### Additional consent provisions for collection and use of participant data and Biological specimens {26b}

All patients will receive a patient information form about the study with a letter of objection they can submit if they do not want to receive questionnaires, reject to have pictures taken and/or object to the processing of their medical data for this study purpose.

### Interventions

#### Explanation for the choice of comparators {6b}

Not applicable.

### Intervention description {11a}

After completion of the mastectomy, a breast field block using bupivacaine or ropivacaine is installed all around underneath the fascia at the edges of the wound. Traditional wound closure consists of closure of subcutaneous tissues using absorbable multifilament 2/0 running suture, and skin closure using 4/0 monofilament resorbable running suture.

The implemented intervention is the quilting suture technique (quilting). With quilting the subcutaneous tissue is sutured to the pectoralis muscle placing multiple rows of running sutures (absorbable, size 0). The suture starts at either end of the scar, running back and forth, creating rows of quilting stiches. The rows are placed transversely from the cranial to the caudal end of the wound with 2–3 cm between them, totalling some three to five rows for the cranial flap. The caudal flap is quilted with two to three rows in a caudal to cranial fashion. The redundant skin is trimmed away, resulting in a tight skin flap, sutured in a tension-free fashion consisting of a subcutaneous absorbable multifilament size 2/0 running suture followed by a 4/0 resorbable monofilament intracutaneous running suture. No wound drain is placed. Prophylactic antibiotics are not warranted, however, can be administered according to local protocol. Topical wound management consists of application of steri-strips. A tight fitting bra or bandage is recommended for the first postoperative days. Regular daily mobilization of the shoulder is stimulated, the first week with shoulder abduction limited to 90 degrees, thereafter without limitation.

### Criteria for discontinuing or modifying allocated interventions {11b}

Subjects can leave the study at any time for any reason if they wish to do so without any consequences. Surgeons can refrain from quilting sutures as deemed necessary or by lack of expertise, after which patients are excluded from participation (no cross over).

### Strategies to improve adherence to interventions {11c}

The quilting technique is a straightforward technique and will be demonstrated and instructed to surgeons by video instructions. On request, surgeons are welcome to join a surgical procedure at the Canisius Wilhelmina Hospital and on site teaching can be provided by an experienced breast surgeon.

### Relevant concomitant care permitted or prohibited during the trial {11d}

Preoperative indication for the operation will be conducted in accordance with the Dutch guidelines, within a frame of mutual shared decision making, supported by a multi-disciplinary team meeting. There are no rules of behaviour for study participants other than described in the “Intervention description” paragraph added to the local protocol following surgery. All concomitant care is permitted during the study, no limitations are imposed.

### Provisions for post-trial care {30}

Not applicable. The quilting suture technique is already a standard surgical technique. Also patients who do not participate in this study (wishing to refrain from filling out questionnaires) are quilted after implementation of the quilting technique in the participating hospitals. The burden of participation consists of filling out questionnaires and the pictures of the breast that are made during follow-up.

### Outcomes {12}

#### Primary study parameter/endpoint

‘Textbook outcome’ (TO), a combination of outcome parameters reflecting an ideal surgical outcome. Measured 6 months post-operative, the patients postoperative course must comply with the following to meet the definition of TO:

- no wound complications
- no re-admissions in relation to primary surgery
- no re-operation in relation to primary surgery, re-excisions in case of involved margins allowed
- no unscheduled visit to the outpatient clinic (depending on the centre one or two postoperative visits are usually scheduled)
- postoperative use of analgesics (6 months) is not increased compared to pre-operative

#### Secondary study parameters/endpoints

Wound complications are defined by the Clavien Dindo classification of Surgical Complications [27] and scored when necessitating (bed side) treatment or follow-up with additional visits to the outpatient clinic, also if beyond 30-days postoperative.

- All palpation-detected seromas (non-aspirated), proportion (%)
- Clinical significant seroma (aspirated seroma), proportion (%)
- Surgical site infections (SSI), proportion (%)
- Wound hematoma or bleeding, skin flap necrosis, wound dehiscence, visualized by inspection, proportion (%)

Health care consumption:

- Duration of surgery: time from incision till wound closed (minutes), mean
- Duration of hospital stay (treatment in day care or amount of nights, day care classified 0), mean
- Numbers of unscheduled visits to the outpatients clinic (one postoperative visit is scheduled), median
- Readmission to the hospital related to primary surgery, median
- Reoperation, related to primary surgery other than re-excision, median

Baseline characteristics based on assumed risk factors for formation of seroma [3, 4, 28–31]:

- Biological gender, proportion (%)
- Age (years at day of surgery), mean
- BMI (kg/m^2^), mean
- Weight of the removed breast (gram), mean
- Polypharmacy (use of five or more medications daily [32], proportion (%)
- TNM classification (type 1 to 4, no malignancy, in situ, ypT0N0, angiosarcoma), nominal, proportion per stage (%)
- Type of surgery (uni- or bilateral mastectomy, ALND or a combination), nominal, proportion per type (%)
- Prior irradiation of the breast in recurrent breast cancer, proportion (%)
- Neoadjuvant chemotherapy, proportion (%)

Other:

- Shoulder function questionnaire (DASH questionnaire = Disability of the Arm, Shoulder and Hand [33], median score
- Post-operative pain (visual analogue scale day 1–14 postoperative and use of analgesics), median score
- Cosmetic outcome (an independent panel of four surgeons will blindly assess cosmetics by classifying standardised digital photographs one a 4-point Likert scale with the following categories: poor, fair, good and excellent. Photos are taken in two positions: the first in neutral position with both arms hanging next to the body and the second with both arms raised in 180gr (or as far as possible) elevation).
- Satisfaction with thoracic wall (BreastQ questionnaire for mastectomy [34]), median score

### Participant timeline {13}

Participation burden consists of filling out questionnaires. The first questionnaires (t = 0 pre-operative) are handed out pre-operatively, (DASH, use of analgesics, BreastQ). Patients are asked to notate the VAS daily during the first two weeks postoperative. Two weeks postoperatively, at scheduled postoperative evaluation, participants will be evaluated by physical examination, a picture of the amputated breast will be taken by the treating surgeon or breast cancer nurse, combined with the DASH questionnaire and use of analgesics again (t = 1). Follow-up lasts for 6 months postoperatively when another picture of the amputated breast is taken and the final DASH and analgesic are completed (t = 2). Additionally, the BreastQ mastectomy module is questioned (Table 1).

**Table 1:** Participant timeline

	**Enrolment**	**Operation**	**Days 1–14 postoperative**	**2 weeks postoperative**	**6 months postoperative**
Patient information form	X				
Letter of objection	X				
Physical examination				X	X
Picture of the thoracic wall				X	X
Questionnaires - DASH - Use of analgesics - Breast Q	X			X	X
VAS pain scores			X		

### Sample size {14}

To calculate the sample size of a randomized controlled trial TO is assumed to be 30% without quilting, as based upon a pilot study [7]. A doubling of TO is considered clinically significant, resulting in a TO of 60% with quilting. In this study 2 treatment arms will be used, with a significance level of 0.05 and power of 80%. For a parallel randomized trial in which *patients* are randomized, a minimum of 44 patients need to be included:

$$2* {\left({\mathrm{Z}}_{\frac{0.05}{2}}+{\mathrm{Z}}_{0.80}\right)}^{2}*\frac{1}{{0.30}^{2}}* 0.45\left(1-0.45\right)=44$$

However, since in the present study *clusters* and not patients are randomized, we need to correct for the fact that outcomes within clusters are very likely more similar than those between clusters. The degree of similarity is estimated using the intra-cluster correlation coefficient (ICC) [35]. This was estimated to be on average 0.028, based upon the pilot study of the 2 hospitals with the variables age, body weight, ASA classification, type of surgery, neo-adjuvant chemotherapy, radiotherapy, tumour classification and first or second episode of breast cancer.

Consequently, the formula as proposed by Hemming et al. was used to correct for this similarity with a total of nine clusters and nine steps [36]. This led to a minimum of 94 patients in total or 11 per centre. A margin safety of 20% was introduced to correct for loss-to-follow-up, which led to a total of 113 patients or 13 per centre. Based on an average of 50 mastectomies each year per centre, the duration of the total inclusion should take 3 months. To correct for the probability of non-adherence to the quilting technique since it is new implemented for all surgeons (safety margin of 20%) each step will take 2 weeks. Suspected transition period and learning curve was suspected to be eight weeks.

### Recruitment {15}

Each hospital assigns a local investigator. The lead investigator maintains contact once every two weeks with each local investigator and monitors recruitment and progress in Castor electronic data capture system (https://www.castoredc.com). Each local investigator monitors local inclusions to make sure all patient receive a patients information and objection form prior to surgery. Furthermore ‘missing inclusions’ are accounted for in the duration of each step and thus the duration of the study (sample size).

### Assignment of interventions: allocation

#### Sequence generation {16a}

At the beginning of the study all hospitals will be randomly assigned to a step. Randomization will be performed with an online randomisation tool by the lead investigator at the beginning of the study.

### Concealment mechanism {16b}

Not applicable.

### Implementation {16c}

Before the start of the study, the lead investigator visits each hospital to inform the professionals involved with breast cancer at the outpatient clinic of the department of surgery of participating hospitals. Implementation of quilting is monitored in each hospital, after randomisation quilting becomes standard practice and all patients undergoing mastectomy and/or axillary lymph node dissection will be quilted, including those individuals who decide not to participate in the study. The quilting technique is a straightforward technique and will be demonstrated and instructed to surgeons by video instructions. On request, surgeons are welcome to join a quilting procedure at the Canisius Wilhelmina Hospital and on site teaching can be provided by an experienced breast surgeon.

### Assignment of interventions: Blinding

#### Who will be blinded {17a}

Not applicable, no blinding.

### Procedure for unblinding if needed {17b}

Not applicable.

### Data collection and management

#### Plans for assessment and collection of outcomes {18a}

Outcome variables are extracted from the electronic patient file by the local investigator.

Baseline characteristics and variables of health care consumption will be collected by data managers.

Data collection forms are constructed in Castor, questionnaires will also be registered in Castor, questionnaires can be sent digital from Castor depending on patients preference.

#### Questionnaires

- DASH [33]
- BreastQ [34, 37].

Pictures of the breast will be taken by the treating surgeon or breast cancer nurse in a standardized fashion. Pictures will be collected by the lead investigator. A panel of four experienced breast surgeons will blindly assess the pictures.

### Plans to promote participant retention and complete follow-up {18b}

The return of questionnaires is monitored by the local and the lead investigator. Participants are contacted by telephone by the local investigator in case of missing questionnaires. Questionnaires can be provided on the outpatient clinic, send digital by mail or a hard copy can be sent by post. Patients will also be contacted by phone if the returned questionnaire does not meet the requirements of assessment.

### Data management {19}

Collected data from the electronic patients files and received questionnaires are processed (coded) into Castor by the local investigator. The key (patient identification list) to the coded data remains safely (locked) in each local hospital and is only accessible for the local investigator. Data extracted from Castor is anonymous and will be handled confidentially and stored not identifiable at the Canisius Wilhelmina Hospital. The collected information will be stored safely for 15 years. The handling of personal data complies with the EU General Data Protection Regulation and the Dutch Act on implementation of the General Data Protection Regulation (in Dutch: Uitvoeringswet AVG, UAVG). The datasets used and/or analysed during the current study are available from the authors on reasonable request.

### Confidentiality {27}

Objected consent forms and collected questionnaires contain personal information. In order to protect confidentiality before, during and after the study these collected papers will be stored safely in an file in a locked room in each local hospital for 15 years.

### Plans for collection, laboratory evaluation and storage of biological specimens for genetic or molecular analysis in this trial/future use {33}

Not applicable.

### Statistical methods

#### Statistical methods for primary and secondary outcomes {20a}

Primary outcome is a binary outcome presented as number of cases and percentage. Nominal data will also be presented as percentage. Continuous data will be presented as mean ± standard deviation or median ± interquartile range, based upon whether the assumption of normality holds (visual interpretation). Analysis of continuous data will be performed by applying the Students T-test for unpaired data in case of continuous variables with a normal distribution. Otherwise the distribution will be compared using the Mann–Whitney U-test. Chi2 test will be used for comparisons of categorical variables between the two groups. The Fisher’s exact test will be performed where applicable. Since data is clustered analysis will be performed using a generalized logistic mixed-effects model with cluster as random intercept. A P-value < 0.05 is considered statistically significant.

### Cost analysis

The cost analysis exists of two main parts. First, volumes of care will be measured prospectively using medical records, resource uses such as visits to the outpatient clinic, medication, hospital (re)admissions and surgical interventions (including intraoperative materials, disposables and operation time). Per arm (Quilt vs drain) full cost-prices will be determined using activity based costing. The second part of the cost analysis consists of determining the cost prices for each volume of consumption in order to use these for multiplying the volumes registered for each participating patient. The Dutch guidelines for cost analyses will be used [38]. For units of care/resources where no guideline or standard prices are available real cost prices will be determined.

Economic analysis will be performed by a specialized statistician.

### Interim analyses {21b}

Not applicable.

### Methods for additional analyses (e.g. subgroup analyses) {20b}

Not applicable.

### Methods in analysis to handle protocol non-adherence and any statistical methods to handle missing data {20c}

Stochastic regression imputation will be used to impute missing data.

Patients lost to follow up will be excluded from the study.

### Plans to give access to the full protocol, participant level-data and statistical code {31c}

Not applicable.

### Oversight and monitoring

#### Composition of the coordinating centre and trial steering committee {5d}

##### Principal Investigator

Design and conduct of the QUILT study.

Preparation of protocol and revisions.

Preparation of patient information forms and questionnaires.

Publication of study reports.

##### Lead Investigator

Design and conduct of the QUILT study.

Preparation of protocol and revisions.

Preparation of patient information forms and questionnaires.

Preparation of Castor.

SUSAR reporting.

Weekly contact with lead investigators on progression.

Publication of study reports.

##### Steering committee (SC)

See title page for members, reviewing publication of study reports.

All local investigators will be steering committee members.

Agreement of final protocol.

Reviewing progress of study, stimulating trial participation and adherence to intervention.

##### Trial Management Committee (TMC)

Principal investigator, lead investigator.

Study planning.

Organisation of steering committee meetings.

Advice for local investigators.

Ethics committee applications.

Data verification.

Data management.

Randomisation.

##### Local Investigators

In each participating centre a lead investigator (nurse practitioner, physician or breast care nurse) will be identified, to be responsible for identification, recruitment, data collection and completion of questionnaires, along with follow up of study patients and adherence to study protocol. Lead investigators will be steering committee members.

### Composition of the data monitoring committee, its role and reporting structure {21a}

A data monitoring committee is not considered necessary since we do not investigate drugs, nor include children or mentally disabled patients, the implemented intervention has no potential to cause life threatening or disabling injuries and we expect to complete the trial in a relatively short period of time.

### Adverse event reporting and harms {22}

Adverse events are defined as any undesirable experience occurring to a subject during the study, whether or not considered related to quilting. All adverse events reported spontaneously by the subject or observed by the investigator or his staff will be recorded.

A serious adverse event is any untoward medical occurrence or effect that at any dose:

- results in death;
- is life threatening (at the time of the event);
- requires hospitalisation or prolongation of existing inpatients’ hospitalisation;
- results in persistent or significant disability or incapacity;
- is a congenital anomaly or birth defect;
- is a new event of the trial likely to affect the safety of the subjects, such as an unexpected outcome of an adverse reaction, lack of efficacy of an IMP used for the treatment of a life threatening disease, major safety finding from a newly completed animal study, etc.

All SAEs will be reported to the accredited METC.

### Frequency and plans for auditing trial conduct {23}

Not planned.

### Plans for communicating important protocol amendments to relevant parties (e.g. trial participants, ethical committees) {25}

Not planned.

### Dissemination plans {31a}

Study data will be communicated to healthcare professionals and the public via peer reviewed publication and presentation at international conferences. Healthcare consumption will be reported in a separate manuscript.

## Discussion

This is the first multicentre prospective study in which quilting without postoperative wound drain will be compared with conventional wound closure with postoperative wound drain following mastectomy and/or axillary lymph node dissection (ALND) with textbook outcome as primary outcome.

To investigate the effect of quilting on quality of health care, a primary outcome parameter is proposed that reflects all relevant parameters and is also suitable for a sample size calculation based on available data from previous studies.

Although often used in previous studies as a primary end-point, seroma can be difficult to define and interobserver variability is likely to occur. Ultrasound could be used to diagnose seroma, however, potential over diagnosis of clinically insignificant small fluid collections would obscure the results. In previous publications the term “clinically relevant seroma” was used to identify those collections necessitating outpatient drainage, often by repeated needle aspiration. This concept is however prone to interobserver subjectivity and physicians’ preference. For these reasons a combined endpoint was defined as an uneventful postoperative course, a so-called textbook outcome following mastectomy and or axillary dissection. Textbook outcome is a multidimensional measure, reflecting an uneventful course following mastectomy and/or ALND. The definition of textbook outcome was established in consultation with surgeons from different hospitals. In this study textbook outcome implies a postoperative course proceeded without wound complications, unscheduled visits to the outpatient clinic, re-admissions, re-operation nor increased use of analgesics compared to pre-operative, measured 6 months post-operative.

The sample size in the protocol is based on data from a previous performed study in two participating hospitals: CWZ and RH [7]. The incidence of textbook outcome was determined from data in both hospitals, where one hospital (CWZ) applied quilting sutures without postoperative drainage as standard practice and the other hospital (RH) used conventional wound closure with postoperative drainage as standard practice.

Surgeons from several hospitals have expressed their interest in learning the technique of quilting, however concerns remain about the impact on overall patient care and health care consumption. Participating in this stepped wedge study enables the combination of implementation of a uniform technique and monitoring the effects of implementation on patient and hospital level. A multicentre individual randomised controlled trial is deemed less suitable for our objectives. Advantage of a stepped wedge design is the immediate implementation of a technique in case of an established positive effect. Furthermore, the learning curve is expected to be steeper compared to alternating the new and old technique. In addition, in the setup of an individual randomised trial patients might deny trial participation and opt for their preferred treatment, either quilting or not. Moreover, the individual randomised trial comes with a higher burden for hospital staff to gain individual informed consent, perform individual randomisation and obtain case report forms. Beside, trial monitoring by investigators is expected to be more time consuming for a randomized trial compared with a stepped wedge study, and thus more expensive. On account of the aforementioned considerations we opted for a stepped wedge study.

While wound closure using quilting sutures is relatively simple, this technique has the potential to change daily clinical practice. We hypothesize that quilting reduces the complication burden of treatment in patients undergoing mastectomy and/or axillary lymph node dissection. We expect the impact on healthcare costs to be beneficial. Prior retrospective data suggest the quilting suture technique takes an additional ten minutes surgery time (estimated cost €160), but shortens hospital stay from inpatient admission to treatment in day care (estimated gain €400), omitting a wound drainage system (€40) and preventing visits to the outpatient clinic for seroma aspiration (€75 per visit with aspiration) [6, 20]. In the Netherlands, where each year 30% of 17.000 women diagnosed with breast cancer undergo mastectomy without immediate reconstruction, this could add up to an annual saving of approximately €1.8 million. Numbers of axillary lymph node dissection, multiple seroma aspirations, impact of surgical site infections, wound dressings, re-admissions or other societal costs such as travelling and work leave among others are not yet taken into account. Therefore, an auspicious beneficial impact is expected in healthcare consumption and thus healthcare costs. However, the magnitude of this budgetary impact remains to be proven in a larger population, preferably over different hospitals.

Previously, concerns have been raised about possible unwarranted side effects of flap suturing following mastectomy and/or ALND. One can imagine that skin flap fixation could impact shoulder range of motion or that multiple sutures can be painful. In case of a thin subcuticular layer, there could be visible skin dimpling at the site of the sutures. In a small prospective dual centre study comparing CWZ and RH, no significant difference between quilted and non-quilted patients was found regarding postoperative shoulder function and postoperative pain and use of analgesics [7]. The QUILT study will report on shoulder function and cosmetic satisfaction from a patients and surgeons perspective in a sufficiently large population. The evaluation will be conceived as extended patient reported outcome measures in the form of standardized and validated questionnaires taken at several points in time. The large patient group and the fact that different institutions and surgical teams participate should enable robust and generalizable conclusions.

Other study protocols regarding postoperative care in breast cancer have been published, such as the SAM trial [39] or the QUISERMAS trial [18]. The SAM trial, a double blind randomized controlled trial, compares flap fixation by quilting sutures with postoperative drainage to conventional wound closure with drainage and to flap fixation by fibrin glue with wound drainage [5]. Flap fixation by sutures following mastectomy leads to a reduction of CSS compared to patients who underwent conventional wound closure or flap fixation by tissue glue. Furthermore unplanned visits were less in the quilted cohort. There were no differences in postoperative shoulder function and cosmetics.

In the QUISERMAS trial, a multicentre, randomized controlled trial, quilting sutures without drainage is compared to conventional wound closure with drainage. In the QUISERMAS trial shoulder function is not assessed by the patient. The range of shoulder movement is reported by the surgeon and cosmetics are assessed by an independent committee. Results of the QUISERMAS trial are awaited.

In conclusion, this study aims to evaluate the effect of implementation of the quilting suture technique in breast cancer surgery. A relatively simple and promising technique that seems effective in reduction of seroma and other wound complications, which are frequently reported following mastectomy and/or ALND. We hypothesize that quilting is a simple technique to decrease the complication burden of treatment and health care consumption. The results of this stepped wedge study will be an important contribution to further explore the quilting technique in order to become an established technique in breast surgery.

## Data Availability

The datasets used and/or analysed during the study are available from the first and senior author on reasonable request.

## Abbreviations

ALND: Axillary lymph node dissection
SSI: Surgical site infection
TO: Textbook outcome
BMI: Body mass index
DASH: Disability of arm, shoulder and hand
VAS: Visual analogue scale
